# Mechanical and Durability Characterization of Hybrid Recycled Aggregate Concrete

**DOI:** 10.3390/ma17071571

**Published:** 2024-03-29

**Authors:** Rashid Hameed, Muhammad Tahir, Safeer Abbas, Haseeb Ullah Sheikh, Syed Minhaj Saleem Kazmi, Muhammad Junaid Munir

**Affiliations:** 1 Civil Engineering Department, University of Engineering and Technology, Lahore 54890, Pakistan; rashidmughal@uet.edu.pk (R.H.); safeer.abbas@uet.edu.pk (S.A.); haseebullahsheikh7@gmail.com (H.U.S.); 2 Department of Civil Engineering, University of Engineering & Technology Lahore, Narowal Campus, Narowal 51601, Pakistan; engrmtahir09@uet.edu.pk; 3 Guangdong Provincial Key Laboratory of Durability for Marine Civil Engineering, Shenzhen University, Shenzhen 518060, China

**Keywords:** concrete, burnt clay bricks, recycled aggregates, hybrid form, mechanical strength, durability, sustainability

## Abstract

The recycling of construction and demolition waste (CDW) for the extraction of recycled concrete aggregates (RCAs) to be used to produce recycled aggregate concrete (RAC) is widely acknowledged internationally. However, CDW not only contains concrete debris but may also contain burnt clay bricks. The recycling of such CDW without the segregation of different components would result in recycled aggregates having different proportions of concrete and brick aggregates. The utilization of these aggregates in concrete requires a detailed investigation of their mechanical and durability properties. In this regard, the present study focused on investigating the mechanical and durability properties of hybrid recycled aggregate concrete (HRAC) made by the 100% replacing of natural aggregates with recycled brick (RBAs) and RCA in hybrid form. The partial replacement of cement with fly ash was also considered to reduce the corban footprint of concrete. An extensive experimental program was designed and carried out in two phases. In the first phase, a total of 48 concrete mixes containing coarse RBA and RCA in mono and hybrid forms were prepared and tested for their compressive strength. The test results indicated that the compressive strength of HRAC is greatly affected by the proportion of coarse RBA and RCA. In the second phase, based on the results of the first phase, eight concrete mixes with the most critical proportions of RBA and RCA in hybrid form were selected to evaluate their mechanical and durability performance. In addition, four mixes with natural aggregates were also prepared for comparison purposes. To evaluate the mechanical properties of the concrete mixes, compressive strength and modulus of rupture (MOR) tests were performed, while for the evaluation of durability properties, water absorption and behavior after exposure to aggressive conditions of acidic and brine solutions were studied. The results revealed that a 20% replacement of cement with fly ash resulted in acceptable mechanical and durability properties of HRAC intended to be used for making concrete bricks or pavers.

## 1. Introduction

Concrete is one of the most widely used construction materials due to its strength, durability, cost effectiveness, and versatility. Conventional concrete is typically a mixture of cement, aggregates, and water, while coarse aggregates contribute to 70% of its volume [1]. Globally, the annual production of concrete is 30 billion tons, which is three times more than the demand for it 40 years ago [2]. Non-renewable natural resources are on the verge of depletion due to the huge production of concrete required for construction activities. In developing countries, besides concrete, burnt clay bricks are also one of the major components of building construction. The brick industry produces 1500 billion bricks each year, out of which, 1300 billion (around 87%) are being manufactured in developing countries [3]. Around 340 billion tons of clay is consumed to manufacture the bricks worldwide [4].

Today, the construction industry is not only the biggest consumer of natural resources but is also the biggest producer of construction and demolition waste (CDW), which is creating environmental pollution and is responsible for approximately 8% of total carbon dioxide emissions globally [5]. CDW is generated because of various natural and manmade activities, such as the demolition of existing buildings, waste generated from the batching plant, waste obtained during the construction and maintenance of huge traffic infrastructure, commercial testing of the concrete in laboratories, and destruction of infrastructure because of catastrophic events such as earthquakes. According to the National Planning Commission of Nepal, around 14 million tons of concrete debris were accumulated within a few days after the 2015 earthquake. Four million tons of waste was found in Kathmandu (the capital city of Nepal) alone, which was equivalent to the total waste generated in 11 years [6]. Around 54,000–87,000 metric tons of municipal solid waste is generated in Pakistan annually [7], out of which around 30% is estimated to be CDW. The annual production of CDW in Pakistan is about 6 million tons [8]. The disposal of CDW is becoming quite expensive because of the shortage of landfill sites, coupled with transportation charges, and it is posing a potential threat to environmental sustainability. Traditionally, CDW is dumped in landfill sites. Since bricks and concrete are the major materials used in the housing construction industry, they contribute to a large portion of CDW. In developing countries, including Pakistan, a huge amount of infrastructural development is expected in the future. In addition, the existing infrastructure is aging and will require replacement. Therefore, a significant volume of CDW is expected to be generated in the near future.

The most effective and sustainable approach to deal with CDW is its recycling to produce recycled concrete aggregates (RCAs) and the utilizing of them as a partial or full replacement of natural aggregates (NAs) in the production of new concrete, commonly known as recycled aggregate concrete (RAC). A life cycle assessment of RCA has revealed that, when CDW is used as RCA, greenhouse gas emissions can be reduced by up to 65% and non-renewable resources can be saved [8,9]. Accordingly, it is logical to overcome the problems associated with CDW by incorporating waste debris as recycled aggregates in concrete. The recycled aggregates can be broadly classified into the following two categories based on the origin of the CDW: RCAs and recycled brick aggregates (RBAs). Due to the inferior properties of RCAs and RBAs, such as higher porosity as compared to NAs, their use in the construction industry on a commercial scale is still limited.

In the recent past, various studies have been conducted to examine the mechanical and durability performance of RAC made by the partial or full replacement of NA with RCA and RBA. For instance, according to Attri et al. [10], the replacement of coarse NA with RCA up to 45%, and fine NA with fine RCA up to 40%, had no significant impact on the performance of paver blocks. According to Ahmed et al. [11], compared to conventionally used burnt clay bricks, the compressive strength and density of RAC bricks were higher, while their water absorption was lower. Further, the performance of RAC bricks was superior to NAC bricks when both were immersed in supersaturated brine and 5% sulfuric acid solutions. As per the study of Farooq et al. [12], the RAC pavers fulfilled the minimum strength (compression and flexure) requirements specified in local and international standards. Further, the RAC pavers were found to be resilient against salt and acid attack. According to Xiao et al. [13], the compressive strength of the specimens prepared with RAC was decreased with the increase in the RCA content. Sasanipour et al. [14] studied the effect of fine and coarse RCAs, with replacement level of 25, 50, 75, and 100%, on the mechanical and durability properties of self-compacting concrete. A decrease in compressive strength was observed for all replacement levels of RCA, while the impact on tensile strength was found to be insignificant. The water absorption and VPV were severely affected by the presence of RCA, and they increased with the increase in the replacement level of RCA. According to Sing et al. [15], the inclusion of fine recycled aggregates resulted in a higher demand of the w/c ratio; however, CO_2_ emission is reduced with the increase of fine recycled aggregates content in the concrete. Reddy et al. [16] investigated the influence of the brick waste as fine aggregates on the properties of recycled aggregate paver blocks. They used brick waste as fine aggregates of a size between 150 µm and 4.75 mm as a replacement for natural fine aggregates by 25, 50, and 75%. Moreover, the fine content of a size < 150 µm in the mix was varied at 10, 20, and 30%. The findings of this study proposed a 50% replacement of natural fine aggregates with brick waste aggregates. However, the replacement can be increased to 75%, provided the fine content is limited to 10% of the mix. According to Mohammed et al. [17], concrete of a strength of 20.7 to 31 MPa can be made using RBA. No significant change in the compressive strength of concrete was found up to a 50% replacement of virgin brick aggregates by RBA. According to Yang et al. [18], it was possible to develop eco-friendly and high-performance recycled lightweight aggregate concrete using a 20% brick-based mineral admixture and 60% brick-based recycled aggregates.

Besides mechanical properties, another important aspect is to study the durability properties of concrete containing recycled clay brick aggregates, as bricks have a porous structure which may lead to a detrimental effect on the durability performance of concrete. In this regard, Ge et al. [19] investigated the durability and shrinkage performance of SCC made using fine RBA, and the results showed that the presence of fine RBA helped to mitigate the drop in the internal relative humidity of SCC during drying, which reduced the risk of drying shrinkage and cracking under restrained conditions. Their study also showed that the use of fine RBA caused a negative effect on the resistance of SCC against chloride ion penetration. According to Dang et al. [20], the replacement of sand with fine RBAs contributed to reducing chloride migration due to the pozzolanic reactivity of RBA; however, their presence caused an increase in carbonation, water sorptivity, water absorption, and drying shrinkage. The pore structure of concrete was observed to be deteriorated in the presence of fine recycled brick aggregates because of their porous structure. Vieira et al. [21] investigated the durability performance of concrete made using fine recycled aggregates from red clay crushed bricks and sanitary ware, and found that in their presence, the water demand of concrete was increased. Further, with the increase in the content of clay brick aggregates in the concrete, its shrinkage was increased. However, the greater modulus of elasticity of sanitary ware aggregates helped to limit the shrinkage of the concrete, which was found to be equal to the control concrete. According to Zong et al. [22], the permeability to chloride ions, air, and water of concrete was increased in the presence of recycled coarse clay brick aggregates. As per the study of Yu et al. [23], the freeze–thaw resistance, resistance to sodium chloride solution, and mechanical strength of concrete was found to gradually decrease with the increasing content of RBA. However, the replacement of cement with fly ash improved the resistance to combined freeze–thaw and chloride attacks. Gayerre et al. [24] investigated the creep and shrinkage in structural concrete incorporating RBA. Natural aggregates were replaced with RBAs by 20, 35, 50, 70, and 100%. The measurement of deformation was performed for 400 days. Their study, based on the values for long-term deformation, concluded that concrete with 20% RBA exhibited a similar response to that of the control concrete.

Recently, research studies have also been carried out in different parts of the world to investigate the properties of concrete incorporating both RCA and RBA. Huang et al. [25] studied the compressive properties of concrete made using RCA and RBA modified with silane (designated as Si-RBA) in mixed form. The results of their study showed that the strengthening of RBAs with 10% silane solution reduced their water absorption by 51.6% and enhanced their crushing index by 14% compared to RCAs. The peak stress and elastic modulus in the compression of concrete was decreased with an increase in ratio between Si-RBA and RCA. Sharma et al. [26] carried out the assessment of the geo-environmental and geotechnical behavior of RCAs, RBAs, and their blends. Based on the low values of impact and crushing resistance of the blends, their application in the surface and base course of pavements was not recommended. Their study suggested that up to 50% RBA in blends may be used for their application in the sub-base and base of pavements, reinforced earth structures, and as fill material in embankments. Liu et al. [27] investigated the effect of coarse RBA content on the mechanical properties of mixed recycled concrete. They found that the compressive strength and modulus of elasticity of concrete incorporating mixed recycled coarse aggregates decreased linearly with an increase in coarse RBA content. The impact of coarse RBA on the flexure and split tensile strength of concrete was observed to be negligible when the total content of coarse RCA and coarse RBA was not more than 30%. Zhang et al. [28] evaluated the performance of a cement-stabilized recycled mixture of RCA and RBA. The impact of the total content of recycled aggregates and the ratio of RBA and RCA on the mechanical properties of concrete was investigated. The result of this study showed that the mechanical properties of the cement-stabilized mixture changed linearly at a recycled aggregates content of 25% and 50% while they sharply decreased when its content was increased from 50% to 75%.

In the past, various research studies have been conducted using brick waste as a base filler or as pozzolanic/supplementary materials [29,30,31,32,33,34,35,36]. However, very few studies are available on the potential use of masonry waste as an RA to prepare concrete. Particularly, research on the use of recycled mixed or hybrid aggregates (both RCAs and RBAs) is very limited. Moreover, in the past two decades, the production of cement has increased three times, from 1.1 billion tons to 3.27 billion tons globally. At the end of 2030, the production of cement will reach up to 4.83 billion tons [26]. Therefore, to provide a sustainable solution for the effective use of construction waste from concrete and masonry structures, a hybrid use of recycled concrete and brick aggregates is proposed to prepare RAC, along with the partial replacement of cement with fly ash. Various mechanical (compressive strength, modulus of rupture, modulus of elasticity) and durability properties (density, water absorption, cyclic ponding in 5% sulfuric acid solution, cyclic ponding in supersaturated brine solution) were studied by preparing HRAC with different replacements of RCA and RBA in hybrid form.

## 2. Materials and Methods

### 2.1. Materials

**Figure 1:**
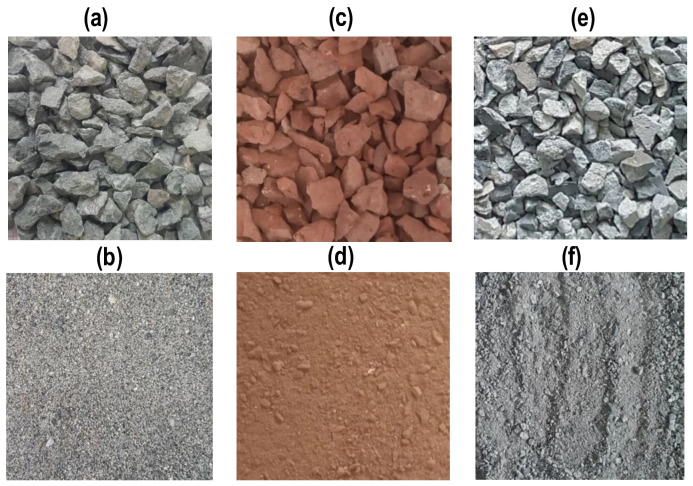
Coarse and fine aggregates; (**a**) natural coarse aggregates; (**b**) natural fine aggregates; (**c**) recycled coarse brick aggregates; (**d**) recycled fine brick aggregates; (**e**) recycled coarse concrete aggregates; (**f**) recycled fine concrete aggregates.

**Figure 2:**
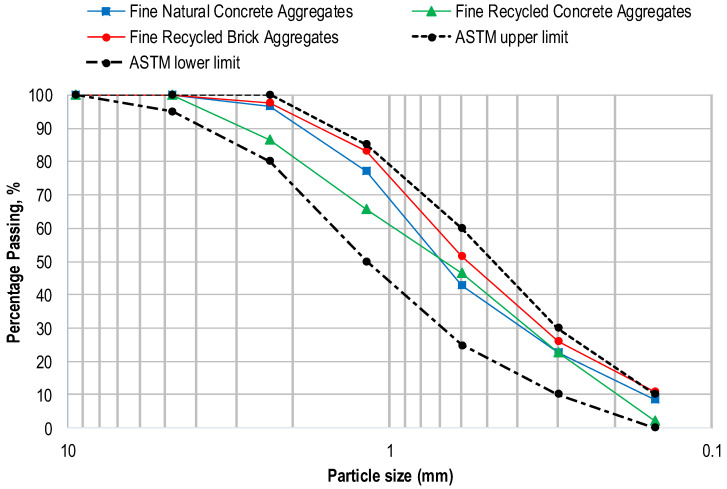
Gradation curves of fine aggregates.

**Figure 3:**
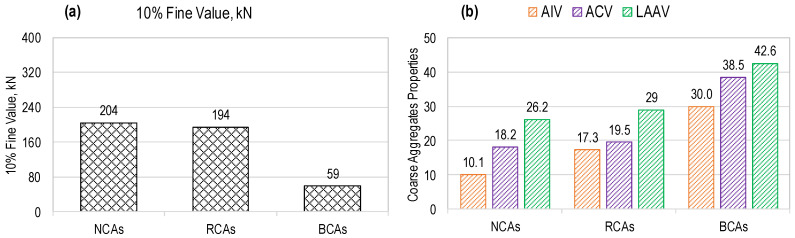
Coarse aggregate properties: (**a**) 10% fine value; (**b**) AIV, ACV, LAAV.

To prepare all the concrete mixes, ordinary Portland cement as a binding material, confirming to the requirements of ASTM C150 [37], and commercially available fly ash as a partial replacement for cement, were used. Two different types of recycled aggregate (RA), RCA and RBA, were used in mono and hybrid form to prepare concrete mixes of RAC. The production process of these aggregates is described in Section 2.2. To prepare mixes of natural aggregate concrete (NAC), locally available crushed stone and river sand were used as coarse and fine NAs, respectively. The coarse and fine fractions of NA, RBA, and RCA are shown in Figure 1. Various tests were performed on fine and coarse aggregates following ASTM/British standards to characterize their physical and mechanical properties, and the results are summarized in Table 1. The maximum size of coarse aggregates used in this work was 12 mm. The gradation curves of three types of fine aggregates are provided in Figure 2, where it is shown that they satisfy the graduation requirements specified in ASTM C33 [38]. The comparison of the mechanical properties of the coarse aggregates is presented in Figure 3. The descending order of aggregates with respect to mechanical properties is NA, RCA, and RBA, as evident from Figure 3. RBA has the least 10% fine value and the highest AIV, ACV, and LAAV, as compared to NA and RCA.

### 2.2. Production of Recycled Aggregates

**Figure 4:**
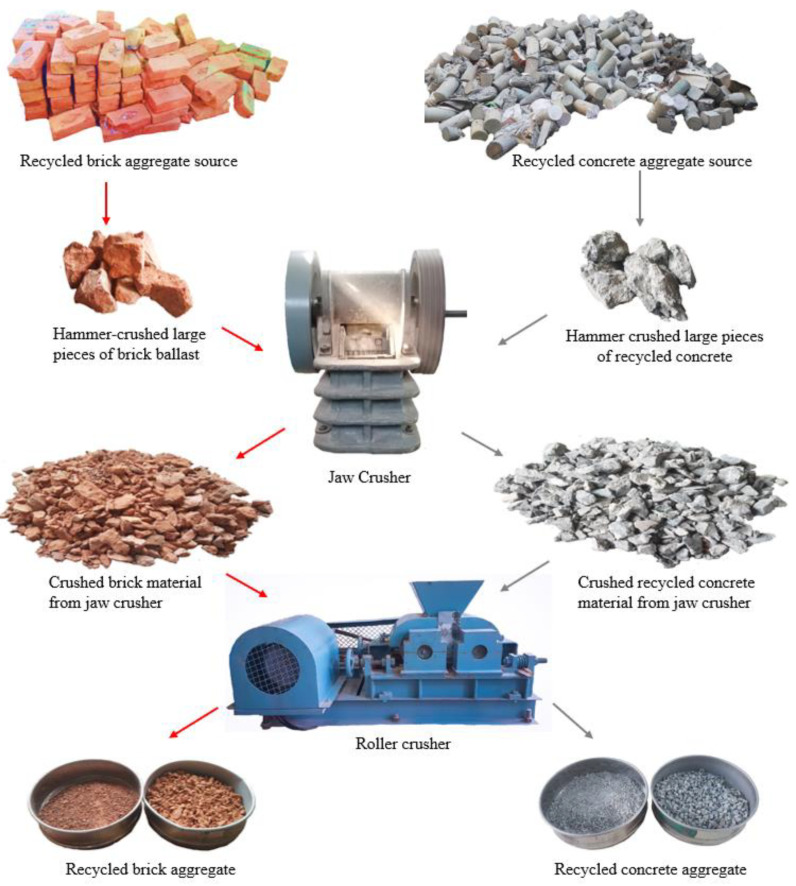
Production process of recycled aggregates.

The complete process of the production of RCA and RBA is shown in Figure 4. As mentioned above, two different types of RA were used in mono and hybrid form to prepare concrete mixes of RAC: RCA and RBA. To produce RCA, commercially tested samples of concrete cubes and cylinders having a compressive strength in the range of 21 MPa to 28 MPa, collected from the concrete testing laboratory, were used.

The waste bricks obtained after their commercial testing were recycled to produce RBA. Initially, tested concrete specimens and bricks were manually broken into pieces of the required sizes to pass them through a jaw crusher, and were then finally passed through a roller crusher to get smaller pieces. At the end, fine and coarse aggregates were separated by sieving.

### 2.3. Mix Proportions

Under the scope of this study, the experimental program was completed in two phases. In Phase 1, a total of forty-eight (48) different types of concrete mixes were prepared to investigate the feasibility of using RBA in mono and hybrid form with RCA to develop RAC. In all mix compositions, the proportion of cement, fine, and coarse aggregate was fixed as 17%, 33% and 50% of the volume of concrete, respectively. The main variables of testing in Phase 1 included the type of fine and coarse recycled aggregates (RCAs and RBAs), the proportion of coarse RCA and RBA in hybrid mixes, and the replacement percentage of cement with fly ash (0%, 20%, 40%, and 60%). The details of the concrete mixes in Phase 1 are presented in Table 2. The water-to-binder ratio for each mix was adjusted (0.5 to 0.7) to achieve the required consistency with respect to vibrated concrete.

Based on the results of the compressive strength of Phase 1, 8 concrete mixes of RAC and 4 concrete mixes of NAC were finally made in Phase 2 to investigate and compare their mechanical and durability performance. The details of all 12 mixes regarding the type of fine and coarse aggregates, and content of cement and fly ash, is provided in Table 3. The nomenclature used in Table 3 is based on the type of fine aggregates, coarse aggregates, and the dosage of fly ash in the mix. For instance, FRB-CHR-20 is for the mix containing 100% fine RBA, coarse RA in hybrid form (60% coarse RBA and 40% RCA), and 20% fly ash (replacement of cement); similarly, FRC-CHR-40 represents a mix containing 100% fine RCA, 60% coarse RBA, 40% RCA, and 40% fly ash (replacement of cement); and FN-CN-60 represents a mix containing 100% fine NA, 100% coarse NA, and 60% fly ash (replacement of cement).

### 2.4. Preparation of Concrete Mix

All concrete constituents were first dry-mixed for one minute, and then water was added along with the mixing until the required consistency of concrete mix was achieved. Considering their high water absorption, both recycled aggregates were used in SSD condition, and water adjustment was made for fine aggregates according to their water absorption capacity. The compaction was performed using a vibrating table, and the demolding of the concrete specimens was performed after 24 h of casting. After demolding, the concrete specimens were cured in water for the next 28 days at a temperature of 24 ± 2 °C.

### 2.5. Specimens Detail and Testing Method

**Figure 5:**
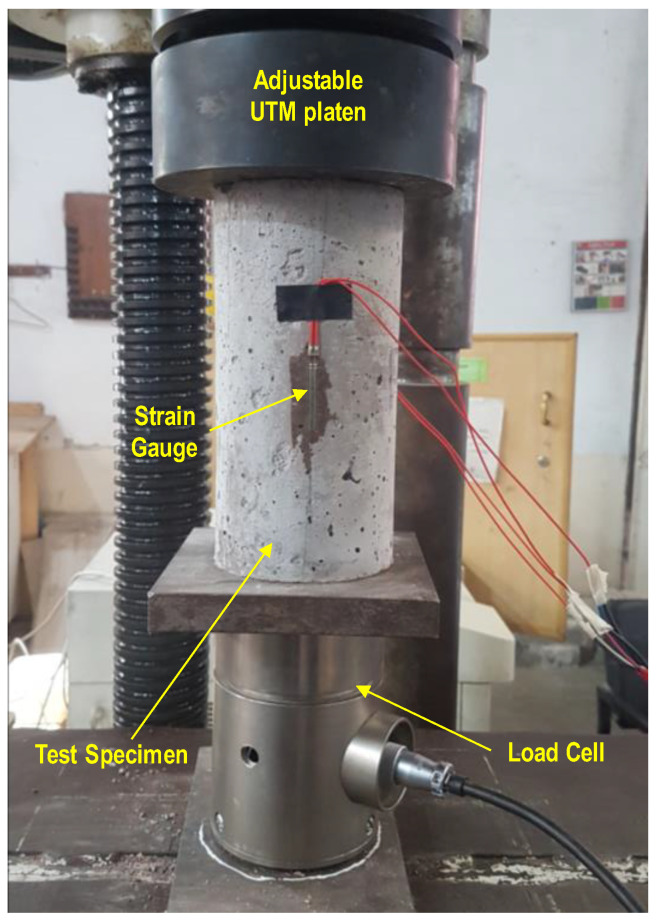
Test setup for compressive strength.

For each mix composition of Phase 1, three cylindrical specimens of 100 mm diameter and 200 mm height were prepared and tested for compressive strength at 28 days. Similarly, for each concrete mix of Phase 2, three cylindrical specimens of 100 mm diameter and 200 mm height (for compressive strength and modulus of elasticity (MOE) in compression), three prismatic specimens of 75 × 75 mm cross section and 300 mm length (for a modulus of rupture (MOR) test), three cubic specimens of size 100 × 100 × 100 mm (for accelerated durability tests), and 2 cubic specimens of the same size (for water absorption tests) were prepared and tested after 28 days of their casting. The compressive strength and MOE were determined following the procedure outlined in ASTM C39 [46] and ASTM C469 [47], respectively. To obtain longitudinal strain, two strain gauges were pasted at 180° on cylindrical specimens, as shown in Figure 5. The MOR was determined as per ASTM C78 [48]. The water absorption test was performed as per ASTM C642 [49]. The accelerated durability tests were performed by the immersion of specimens in 5% sulfuric acid and brine solutions, following the procedure adopted by Ali et al. [50].

## 3. Results and Discussion

### 3.1. Compressive Strength: Phase I Testing

**Figure 6:**
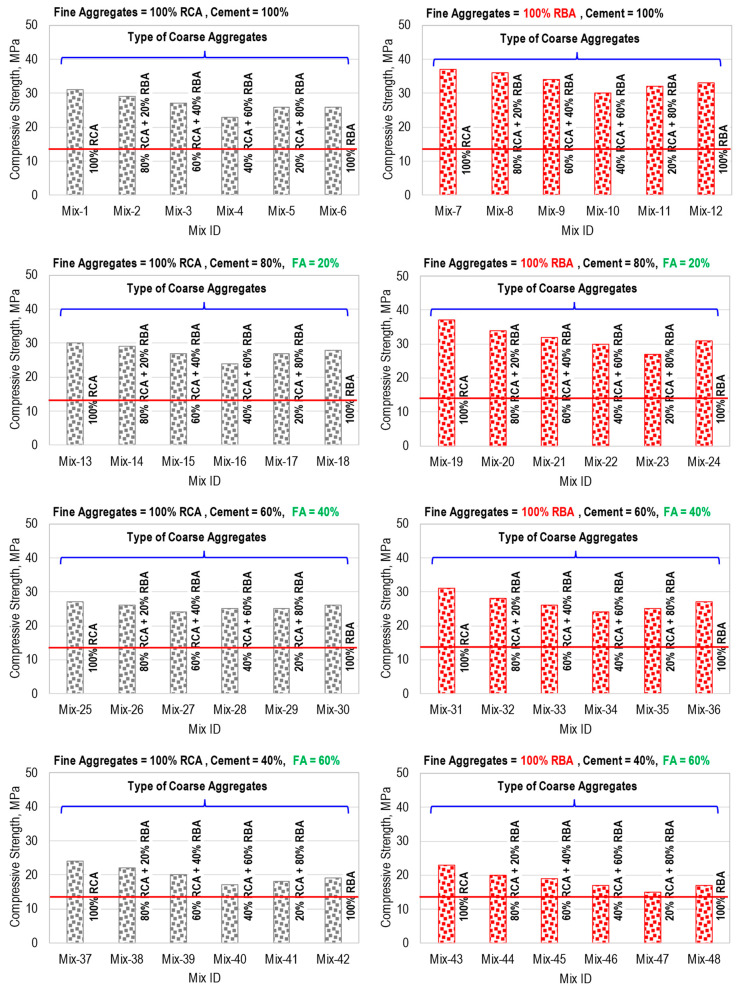
Compressive Strength (Phase 1 testing).

The compressive strength results of the 48 concrete mixes tested in Phase 1 are presented in Figure 6. The results showed that the type of fine aggregates, the type and proportion of coarse aggregates in the hybrid mix, and the fly ash content affected the compressive strength of HRAC.

**Figure 7:**
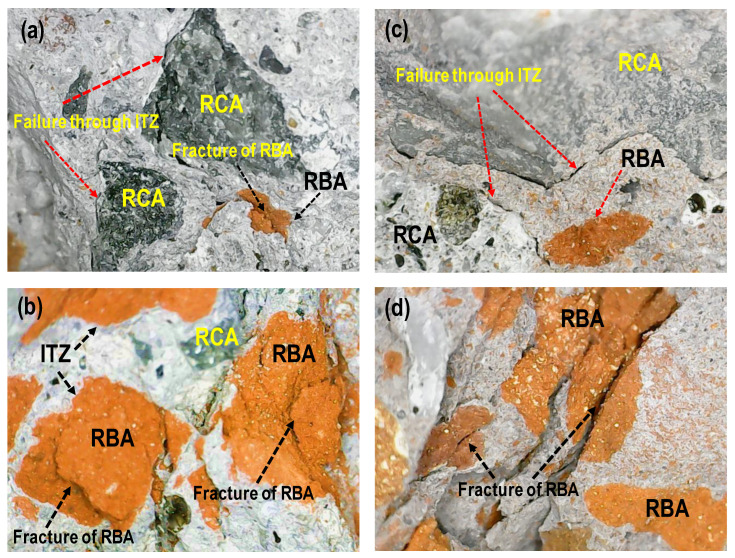
Failure modes in compression of HRAC (Phase 1): (**a**) Mix-2; (**b**) Mix-5; (**c**) Mix-8; (**d**) Mix-11.

From the results of mixes M-1 to M-6 and mixes M-7 to M-12, it is clear that the compressive strength of HRAC decreased as the percentage content of coarse RBA increased up to 60%, irrespective of the type of fine aggregate. A similar observation was made by [51]; however, strength increased with a further increase of the coarse RBA percentage up to 100%. However, it remained less than the compressive strength of RAC containing 100% coarse RCA. A similar trend can be noticed for the concrete mixes M-9 to M-48. This is mainly attributed to the fact that the coarse RBAs have a lesser strength as compared to coarse RCA, and the failure of the concrete occurred through particles of coarse RBA, instead of the cement matrix or ITZ, as is evident from Figure 7. The results showed that, among the mixes containing 100% fine RCA, Mix-40 exhibited the least compressive strength, while among the mixes containing 100% fine RBA, Mix-47 attained the minimum compressive strength.

The comparison of the compressive strength of the mixes M-1 to M-12 indicated that the use of fine RBA in HRAC improved the strength of the concrete, as compared to the concrete mixes with fine RCA; a similar observation was made by [52]. The lesser strength in the case of fine RCA may be attributed to the presence of weaker cement paste in HRAC. However, the use of fine RBAs demanded a higher w/c ratio because of their higher water absorption value, as is evident from the results of their physical properties presented in Table 1. The comparison of the results of all 48 mixes presented in Figure 6 indicated that, with an increase in cement replacement percentage with fly ash, the compressive strength of RAC reduced. For instance, the concrete mixes M1, M13, M25, and M37 with a 0, 20%, 40%, and 60% replacement of cement with fly ash, exhibited a compressive strength of 31, 30, 27, and 24 MPa, respectively. It is worth noting that the compressive strength of all mix proportions prepared and tested in Phase 1 was greater than the minimum target compressive strength of 13.4 MPa, which satisfies the compressive strength requirement for loadbearing concrete masonry units specified in ASTM C90 [53] and ASTM C55 [54].

Keeping in view the objective of this study related to the development of sustainable concrete to be used in making bricks and paving blocks, the most suitable mixes were selected for further study. A concrete mix with 40% coarse RCA and 60% coarse RBA attained the least strength out of all the mixes and was selected for further study, keeping in view the most extreme possible proportion of aggregates that can be obtained from the debris. Similarly, the replacement of cement with fly ash was also considered, despite the negative impact on strength, in order to prepare sustainable concrete with the least possible amount of cement. The details of selected mixes are presented in Table 3.

### 3.2. Results of Mechanical and Durability Properties (Phase 2)

The experimental program of the second phase was designed to investigate the mechanical and durability properties of HRAC. Various tests, including density, compressive strength, modulus of elasticity, flexural strength, water absorption, cyclic ponding in 5% sulfuric acid solution, and cyclic ponding in supersaturated brine solution, were performed. The results of the tests are presented below.

#### 3.2.1. Density

**Figure 8:**
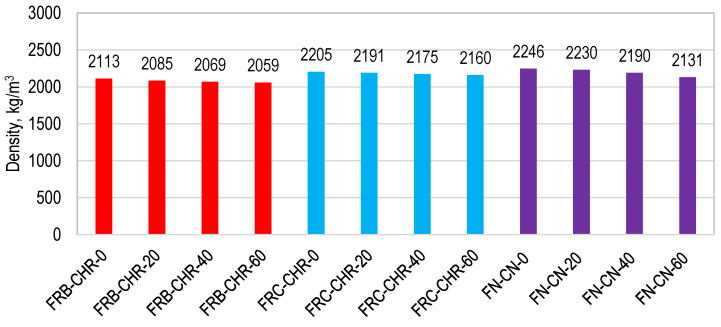
Density of HRAC and NAC specimens.

The density of all the concrete mixes is shown in Figure 8. FRB-CHR-0, FRC-CHR-0, and FN-CN-0 achieved a density of 2113, 2205, and 2246 kg/m^3^, respectively. These results indicated that the density of NAC samples was greater than density of HRAC samples because of the high bulk density of NA compared to RCA and RBA. However, the density of HRAC specimens containing fine RCA was more than HRAC specimens containing fine RBA. This was because of the high bulk density of fine RCA as compared to fine RBA, as shown in Table 1. A similar trend of density was observed for other specimens having a cement replacement percentage of 20, 40, and 60%.

The density of concrete decreased with an increase in fly ash content for all concrete mixes containing RA and NA, and this is mainly attributed to the fact that heavier particles of cement are replaced with lighter particles of fly ash. The results also indicated that HRAC containing fine RBA exhibited a lower density than that of concrete containing fine RCA or NA, due to the comparatively lower specific gravity of RBA.

#### 3.2.2. Compressive Strength

**Figure 9:**
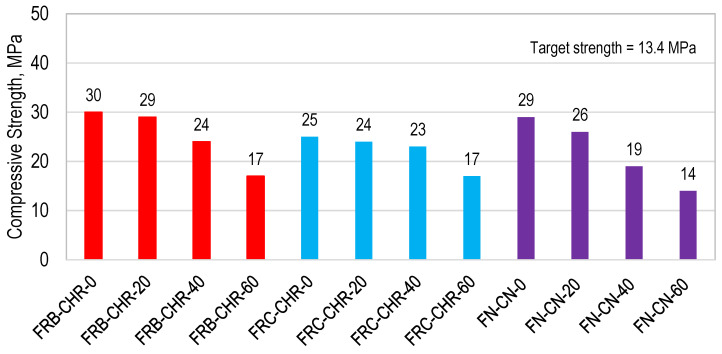
Compressive Strength of HRAC and NAC specimens.

Compressive strength results are shown in Figure 9. A maximum compressive strength of 30, 25, and 29 MPa was developed by FRB-CHR-0, FRC-CHR-0, and FN-CN-0, respectively, containing 100% cement as a binding material. Maximum strength was achieved by HRAC containing fine RBA, compared with HRAC containing fine RCA and NAC. The higher strength in the case of fine RBA is due to the maximum filling effect provided by these aggregates due to their fineness, as observed in the results of Phase 1 of this study. Similar results were obtained by [55]. The HRAC specimens containing fine RBA developed a compressive strength of 30, 29, 24, and 17 MPa for a fly ash content of 0, 20, 40, and 60%, respectively. The compressive strength of the concrete decreased with the increase in the replacement level of cement with fly ash. A similar trend was observed for HRAC containing fine RCA and NAC. When cement was replaced up to 60% with fly ash, a minimum compressive strength of 17 MPa, 17 MPa, and 14 MPa was achieved by HRAC containing fine RBA, fine RCA, and NAC, respectively, which was greater than the target strength of 13.4 MPa. From these observations and the test results, it can be concluded that the use of recycled aggregate in hybrid form can develop strength more than the minimum required for making concrete masonry units with fine and coarse NA, RCA, or RBA, keeping the cement replacement level lesser than or equal to 60%. The results for compressive strength were consistent with the results for density; a higher density of concrete results in higher strength. The above-presented results for compressive strength showed that the replacement of cement with fly ash could result in sustainable concrete with acceptable mechanical properties.

#### 3.2.3. Modulus of Elasticity

**Figure 10:**
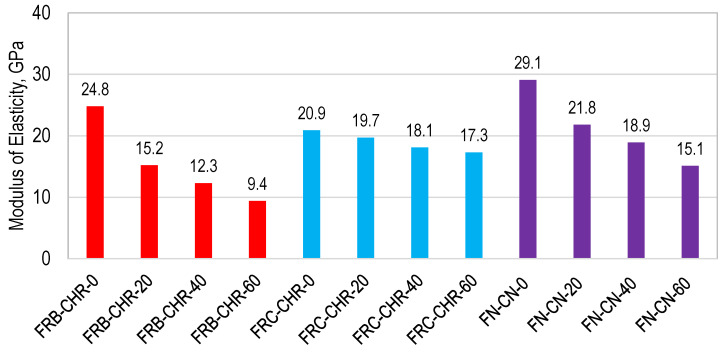
Modulus of elasticity of HRAC and NAC specimens.

The modulus of elasticity of HRAC specimens is shown in Figure 10. It was observed that the maximum MOE of all concrete mixes was achieved when 100% cement was used as a binding material. In addition, the MOE of HRAC with fine RBA was higher than the one achieved with fine RCA at 0% replacement of cement, but it was lesser when the cement was partially replaced with fly ash. However, NAC achieved the highest MOE compared to HRAC with fine RCA and fine RBA, and it can be attributed to the porous nature of recycled materials compared to NA. In the case of NAC and HRAC, the MOE decreased with the increase in the replacement ratio of cement with fly ash.

#### 3.2.4. Flexural Strength

**Figure 11:**
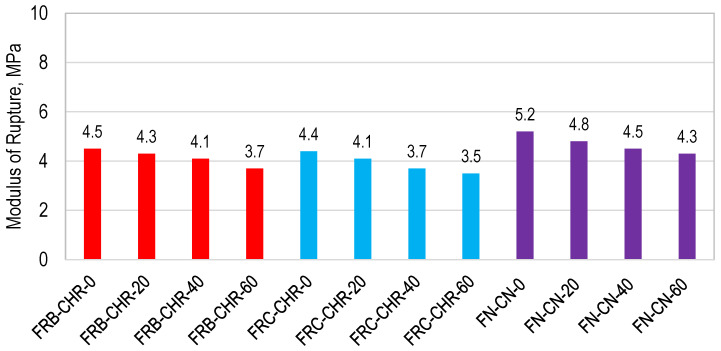
Modulus of rupture of HRAC and NAC specimens.

The modulus of rupture (MOR) of all concrete specimens is shown in Figure 11. A maximum value of MOR of 4.5, 4.2, and 5.2 MPa was achieved by HRAC containing fine RBA, fine RCA, and NAC, respectively, when 100% cement was used as a binding material. Considering the replacement of cement with fly ash, the highest MOR was obtained at a 20% replacement level for all aggregate proportions. This indicated the negative impact of cement replacement on the MOR of concrete, similar to that of compressive strength and density. The results showed that the mix of HRAC containing fine RBA achieved a higher MOR, as compared to HRAC with fine RCA.

From the above results of mechanical properties, it can be concluded that the most suitable replacement percentage of cement with fly ash is 20% for HRAC as well as NAC, producing a sustainable concrete mix able to develop the required mechanical properties. Similarly, the use of fine RBAs in HRAC is comparatively more suitable, as they resulted in better mechanical properties compared to fine RCAs.

#### 3.2.5. Water Absorption

**Figure 12:**
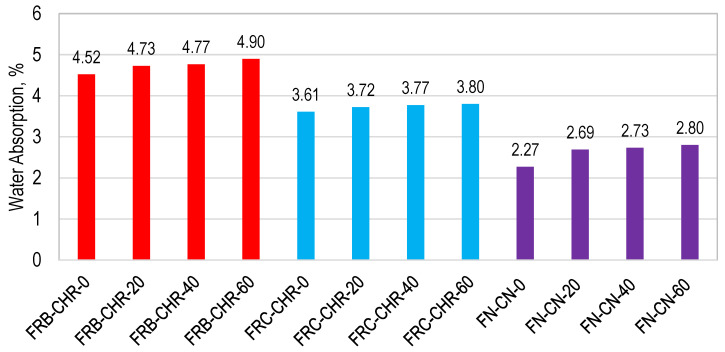
Water absorption of HRAC and NAC specimens.

The water absorption (WA) capacity of concrete is a direct measure of its porosity and also affects its durability properties. The WA of concrete can also be linked with mechanical properties like compressive strength. The results of WA of all concrete specimens are shown in Figure 12. A water absorption of 4.52, 3.61, and 2.27% was exhibited by HRAC containing fine RBA, fine RCA, and NAC, respectively, at a 0% replacement of cement with fly ash. This is due to the lesser porosity and water absorption capacity of both coarse and fine NAs, as compared to RCA and RBA. The HRAC specimens containing fine RBA showed the maximum water absorption, due to the high porosity and water absorption capacity of fine RBA as compared to fine RCA and NA. However, it was observed that cement replacement resulted in an increase in the water absorption of concrete specimens, irrespective of the type of fine and coarse aggregates. The higher water absorption indicated an increase in the porosity of the concrete with the replacement of cement with fly ash and is consistent with the results of the density and compressive strength of the concrete presented above.

#### 3.2.6. Cyclic Ponding in 5% Sulfuric Acid Solution

**Figure 13:**
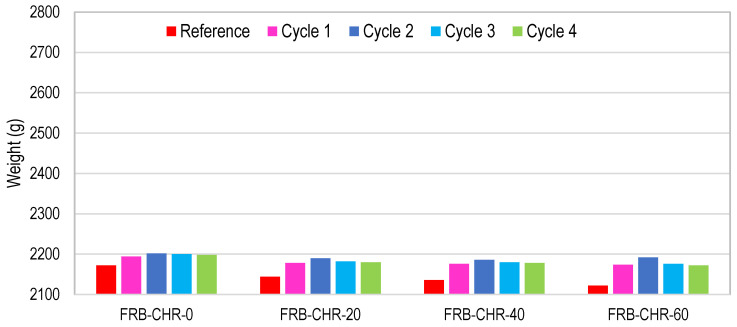
Mass change of HRAC with fine RBA during four cycles of acid immersion.

**Figure 14:**
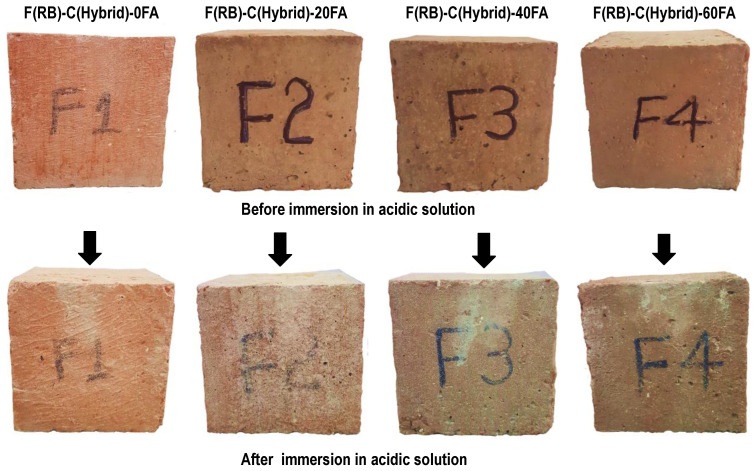
Gypsum formation on the surface of HRAC with FRBA after four cycles of acid immersion.

To perform this test, concrete samples were immersed in a 5% dilute sulfuric acid solution for a duration of 3 days and were dried at normal atmospheric conditions for the next 4 days. The whole wetting and drying cycle lasted for one week, and a total of four such cycles were performed. The change in the mass of the concrete specimens was recorded, and the formation of efflorescence on the surface of the specimens and the cracking or scaling of the concrete were observed. In HRAC specimens with fine RBA, the mass of the HRAC samples increased significantly after one cycle due to the high porosity of fine RBA, as shown in Figure 13. In HRAC specimens containing fine RBA, it was noticed that, when the fly ash content was increased from 0% to 60%, the gypsum (whitish powder) formation on the surface of the HRAC samples was reduced, as shown in Figure 14. In HRAC containing fine RBA, the maximum increase in mass was 1.2, 2.1, 2.3, and 3.3% due to acid immersion for a 0, 20, 40, and 60% replacement of cement with fly ash. The change/increase in mass increased with the increase in fly ash content; this is in line with the water absorption capacity results presented earlier. No deterioration was observed on the surface of samples after four cycles of acid immersion. This shows that the HRAC containing fine RBA has a good resistance against an acidic environment. Similarly, the replacement of cement with fly ash improved the resistance against an acidic environment.

**Figure 15:**
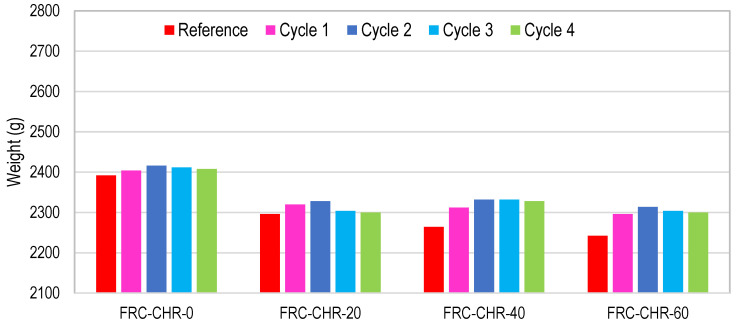
Mass change of HRAC with fine RCA during four cycles of acid immersion.

**Figure 16:**
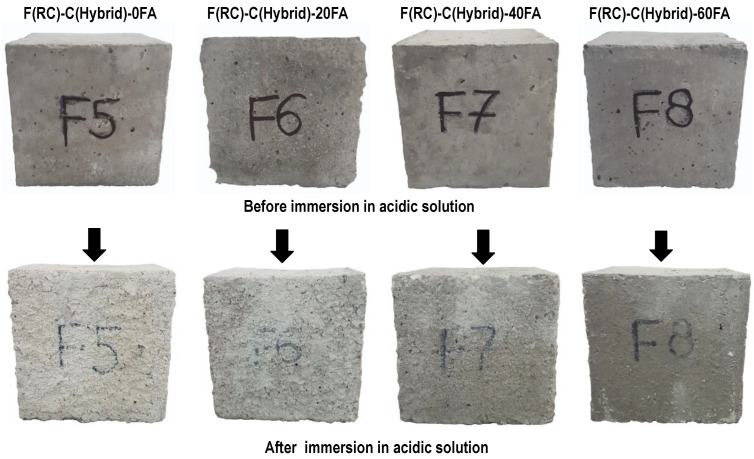
Gypsum formation on the surface of HRAC with fine RCA after four cycles.

In HRAC containing fine RCA, the maximum mass was increased up to 1.0, 1.4, 3.0, and 3.2% due acid immersion for a 0, 20, 40, and 60% replacement of cement with fly ash, respectively, as shown in Figure 15. In almost all of the mixes, the mass increased up to the second cycle of immersion, and for the latter cycles, it started to reduce due to the scaling of the concrete. The change in the mass of HRAC containing fine RBA was more than that for HRAC incorporating fine RCA, due to the higher porosity of concrete containing fine RBA. In HRAC specimens where FRCA was used, both gypsum formation and surface deterioration occurred side by side. Severe and dense form of whitish powder formed on the surface of the HRAC; however, when more cement was replaced with fly ash, the whitish powder on the surface of samples started to reduce, as shown in Figure 16. The cement matrix of RCA contains more hydrated products, so fine RCA showed more gypsum formation as compared to fine RBA in HRAC mixes.

**Figure 17:**
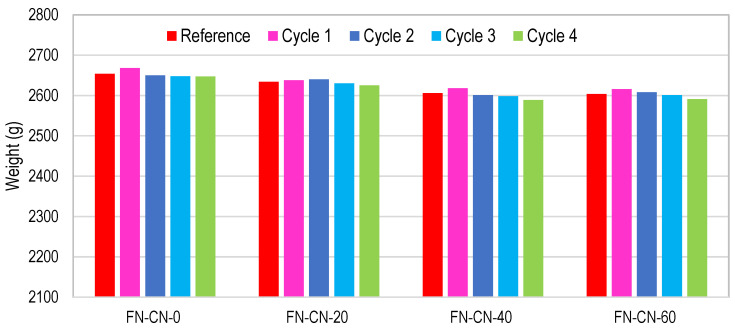
Mass change of NAC during four cycles of acid immersion.

**Figure 18:**
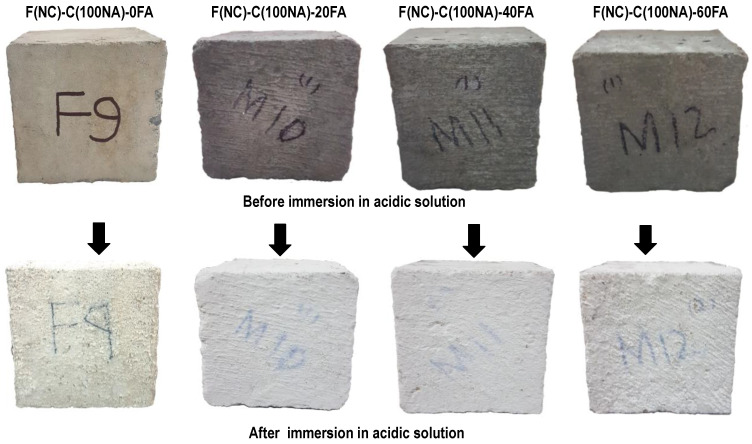
Gypsum formation on the surface of NAC specimens after four cycles.

In NAC specimens, the mass of the concrete specimens increased slightly after one cycle, as shown in Figure 17. It was observed that, after the completion of four cycles of acid immersion, the weight of all NAC specimens decreased by up to 0.6%. Both surface deterioration and gypsum formation occurred on the surface of the concrete samples, but the intensity of whitish powder was lower, as compared to HRAC specimens containing fine RCA, as shown in Figure 18.

**Figure 19:**
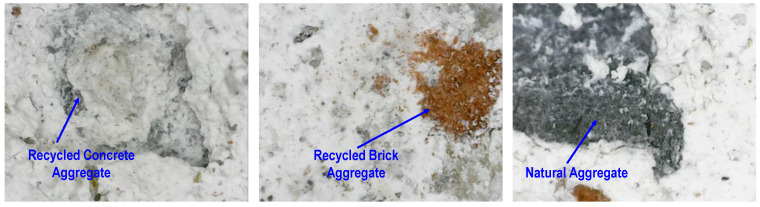
Gypsum formation on the surface of concrete specimens after four cycles.

Magnified images of the surface of the specimens after the completion of four cycles of acid immersion are shown in Figure 19. It can be seen that, due to adhered mortar on the surface of RCA, gypsum formation also occurred on RCA, along with the cement matrix. However, less gypsum formed on the surface of coarse RBA and NA. This is attributed to the fact that the NAs were inert and did not react with the sulfuric acid. This observation indicates that the use of RBA in concrete can yield durable concrete in an acidic environment.

**Figure 20:**
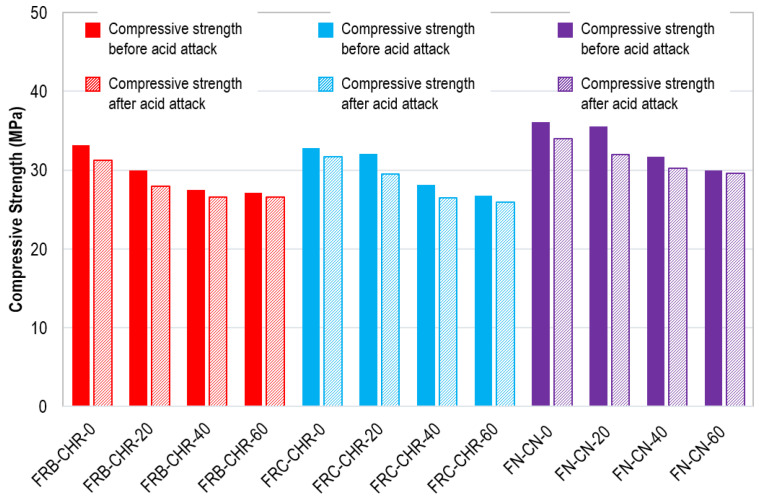
Compressive strength before and after acid immersion.

After the completion of four cycles of acid immersion, a compressive strength test was performed on all concrete specimens. The compressive strength of all the concrete specimens before and after acid attack is shown in Figure 20. In general, the compressive strength of the concrete specimens decreased after acid attack. The loss of compressive strength was greater for concrete specimens containing 100% cement. However, when cement replacement with fly ash was increased, the decreasing rate of compressive strength was also reduced. This was because of the fact that sulfuric acid reacted more with cement than with fly ash. These observations indicated that the use of fly ash as a replacement for cement helps to improve the durability of concrete.

#### 3.2.7. Cyclic Ponding in Supersaturated Brine Solution

**Figure 21:**
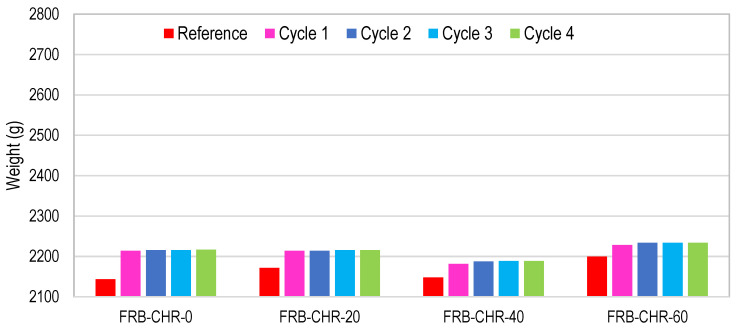
Mass change of HRAC with fine RBA during four cycles of salt immersion.

**Figure 22:**
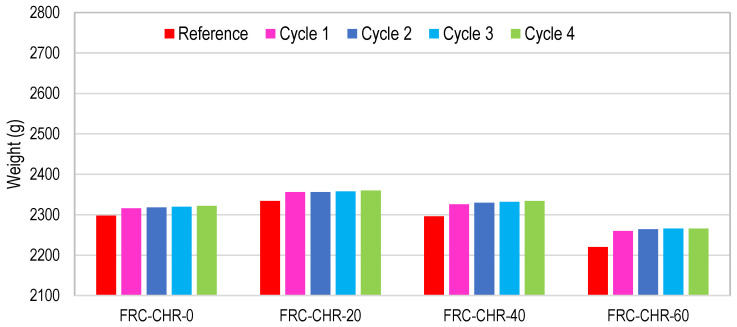
Mass change of HRAC with fine RCA during four cycles of salt immersion.

**Figure 23:**
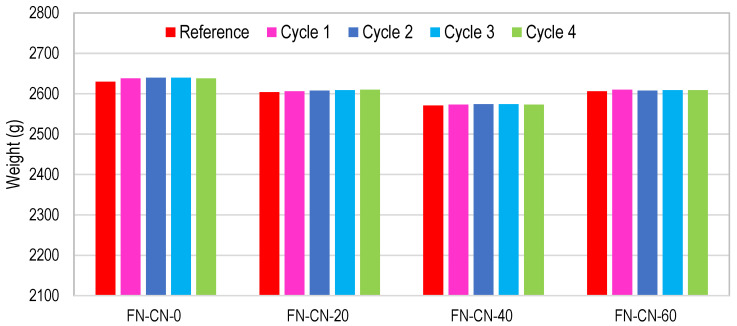
Mass change of NAC with NAs during four cycles of salt immersion.

For cyclic ponding in a supersaturated brine solution, concrete cubes were immersed in brine solution for a period of 7 days and were allowed to dry at normal room temperature for the next 7 days. One cycle of wetting and drying for this test consisted of 14 days (2 weeks), and a total of four such cycles of wetting and drying were performed. In HRAC specimens where fine RBA was used, the mass of HRAC samples increased significantly after one cycle, as compared to the mass of HRAC containing fine RAC, due to the high porosity of fine RBA as compared to fine RCA, as shown in Figure 21 and Figure 22. Afterwards, the mass difference of HRAC specimens containing fine RBA was negligible. Less efflorescence was observed on the surface of HRAC specimens with fine RBA as compared to HRAC specimens with fine RCA. In NAC specimens, the change in mass after the completion of four cycles was not significant, as shown in Figure 23.

**Figure 24:**
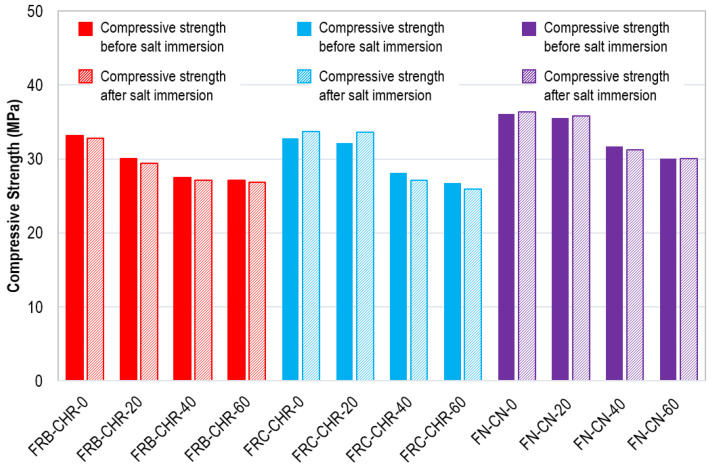
Compressive strength before and after salt immersion.

After the completion of four cycles of supersaturated brine solution immersion, a compressive strength test was performed on all concrete specimens. The compressive strength of all concrete specimens before and after salt immersion is shown in Figure 24. It was observed that, in the case of HRAC with fine RBA, the difference in the compressive strength of specimens before and after immersion was negligible. This is because the HRAC with fine RBA were less affected by the brine solution. Moreover, in HRAC with fine RCA and NAC specimens, the strength of the concrete specimens was slightly improved due to salt crystallization, which strengthens the concrete specimens by densifying their cement matrix. However, when more cement content was replaced with fly ash, less salt crystallization occurred, and the strength was decreased.

Exposure of HRAC containing fine RBA to the saline environment exhibited acceptable performance, just like in an acidic environment. Hence, it can be concluded from these observations that the use of fine RBA in HRAC is beneficial in terms of mechanical and durability performance and an economic perspective.

## 4. Conclusions and Recommendations

This study aimed to investigate the mechanical and durability properties of hybrid recycled aggregates concrete. The study was completed in two phases: in the first phase, the feasibility of using recycled brick aggregates in hybrid form with recycled concrete aggregate to prepare recycled aggregate concrete was studied; and in the second phase, the mechanical and durability properties of selected concrete mixes were studied in detail. The main study parameters included the type of fine aggregate, the proportion of recycled brick and concrete aggregates, and the replacement level of cement with fly ash. Based on the test results and observations, the following conclusions have been drawn:

- Due to the higher bulk density of fine RCA as compared to fine RBA, the HRAC containing fine RCA exhibited the maximum density. This showed that RAC with lower weight can be designed using fine BRA.
- Although the compressive strength and MOE of RAC decreased with the replacement of cement with fly ash, a replacement level up to 60% resulted in a compressive strength satisfying the minimum requirement of 13.4 MPa.
- The compressive strength of hybrid recycled aggregate concrete is greatly affected by the proportion of coarse recycled brick and concrete aggregates. The least compressive strength was achieved when 60% of coarse RBAs were used, along with 40% of coarse RCAs.
- The 20% replacement of cement with fly ash resulted in acceptable mechanical and durability properties of concrete intended to be used for making concrete bricks, along with a reduction in cement content which is a source of CO_2_ emissions. Hence, strong, durable as well as relatively green concrete can be designed using a 20% replacement of cement with fly ash.
- The modulus of rupture of HRAC was affected by the type of fine aggregate; the least value of MOR was exhibited by HRAC containing fine RBA and fly ash as a partial replacement for cement.
- The use of fine recycled brick aggregates helped to improve the compressive strength and durability of concrete, as compared to fine recycled concrete aggregates; however, it had a negative impact on the water absorption capacity of concrete.

Finally, it is concluded that CDW containing clay bricks and concrete can be recycled to produce RAC to be used for the manufacturing of concrete brick units with desirable mechanical properties, which will result in durable and sustainable construction.

As this study was aimed at designing recycled aggregate concrete with RCA and RBA to be used in the production of masonry units, further study may be carried out to investigate the corrosion behavior of steel bars embedded in such concrete, keeping in view its application in reinforced concrete structural members.

## Figures and Tables

**Table 1:** Properties of fine and coarse aggregates.

Properties	Testing Standard	NAs		RCAs		RBAs	
		Coarse	Fine	Coarse	Fine	Coarse	Fine
Bulk oven-dry specific gravity	ASTM C127 [39] and ASTM C128 [40]	2.7	2.58	2.21	2.05	2.1	-
Water absorption (%)	ASTM C127 [39] and ASTM C128 [40]	1.9	2.3	8.7	10.4	13.5	16.1
Rodded bulk density (kg/m^3^)	ASTM C29 [41]	1532.6	1654.6	1321	1426.3	1310	1390
10% fine value, (kN)	BS 812-111 [42]	204	-	194	-	59	-
Aggregate impact value (AIV), (%)	BS 812-112 [43]	10.1	-	17.3	-	30.0	-
Aggregate crushing value (ACV), (%)	BS 812-110 [44]	18.2	-	19.5	-	38.5	-
Los Angeles abrasion value (LAAV), (%)	ASTM C131 [45]	26.2	-	29	-	42.6	-

**Table 2:** Details of mix proportions for Phase 1 testing.

Mix ID	Cement (Volume Fraction)	Fly Ash (Volume Fraction)	Fine Aggregates (Volume Fraction, V_f_)		Coarse Aggregate (Volume Fraction, V_f_)	
			RCA	RBA	RCA	RBA
M-1	17%	----	33%	----	50%	----
M-2					40%	10%
M-3					30%	20%
M-4					20%	30%
M-5					10%	40%
M-6					----	50%
M-7	17%	----	----	33%	50%	----
M-8					40%	10%
M-9					30%	20%
M-10					20%	30%
M-11					10%	40%
M-12					----	50%
M-13	13.6%	3.4%	33%	----	50%	----
M-14					40%	10%
M-15					30%	20%
M-16					20%	30%
M-17					10%	40%
M-18					----	50%
M-19	13.6%	3.4%	----	33%	50%	----
M-20					40%	10%
M-21					30%	20%
M-22					20%	30%
M-23					10%	40%
M-24					----	50%
M-25	10.2%	6.8%	33%	----	50%	----
M-26					40%	10%
M-27					30%	20%
M-28					20%	30%
M-29					10%	40%
M-30					----	50%
M-31	10.2%	6.8%	----	33%	50%	----
M-32					40%	10%
M-33					30%	20%
M-34					20%	30%
M-35					10%	40%
M-36					----	50%
M-37	6.8%	10.2%	33%	----	50%	----
M-38					40%	10%
M-39					30%	20%
M-40					20%	30%
M-41					10%	40%
M-42					----	50%
M-43	6.8%	10.2%	----	33%	50%	----
M-44					40%	10%
M-45					30%	20%
M-46					20%	30%
M-47					10%	40%
M-48					----	50%

**Table 3:** Details of mix proportions for strength and durability study.

Mix ID	Designation	Cement	Fly Ash	Type of Fine Aggregate & V_f_	Coarse Aggregate, V_f_		
					RBAs	RCAs	NAs
M-1	FRB-CHR-0	17%	----	RBAs & 33%	30%	20%	0%
M-2	FRB-CHR-20	13.6%	3.4%		30%	20%	0%
M-3	FRB-CHR-40	10.2%	6.8%		30%	20%	0%
M-4	FRB-CHR-60	6.8%	10.2%		30%	20%	0%
M-5	FRC-CHR-0	17%	----	RCAs & 33%	30%	20%	0%
M-6	FRC-CHR-20	13.6%	3.4%		30%	20%	0%
M-7	FRC-CHR-40	10.2%	6.8%		30%	20%	0%
M-8	FRC-CHR-60	6.8%	10.2%		30%	20%	0%
M-9	FN-CN-0	17%	----	NAs & 33%	0%	0%	50%
M-10	FN-CN-20	13.6%	3.4%		0%	0%	50%
M-11	FN-CN-40	10.2%	6.8%		0%	0%	50%
M-12	FN-CN-60	6.8%	10.2%		0%	0%	50%

## Data Availability

Data are contained within the article.
